# Supplementary material for: Predicting Visit Cost of Obstructive Sleep Apnea Using Electronic Healthcare Records With Transformer
Source: IEEE J Transl Eng Health Med. 2023 May 17;11:306–17. doi: 10.1109/JTEHM.2023.3276943 (PMC10234513; doi:10.1109/JTEHM.2023.3276943)
Supplement: Supplementary materials [file supp1-3276943.pdf]

Predicting Visit Cost of Obstructive Sleep Apnea using Electronic Healthcare Records with Transformer

Zhaoyang Chen, Lina-Siltala Li, Mikko Lassila, Pekka Malo, Eeva vilkkumaa, Tarja Saaresranta, Arho Veli Virkki

Supplementary information:

Legend:

Table S1: Description of the whole OSA cohort.

Table S2: Description of the OSA cohort for cost prediction.

Figure S1. Visit cost distribution.

Figure S2. Age distribution for both genders.

Figure S3. Visit cost distribution for each visit type.

Figure S4. Visit cost distribution for each department.

Figure S5. Annual number of new diagonsis for both genders.

Figure S6. Age distriubution at each year of diagnosis.

Figure S7. Annual visit frequency for each specialist.

Figure S8. Three common logarithm functions.

Table S3: Model performance based on test data.

Figure S9.Training and validation RMSE curves for model 3, 7, and 11.

Figure S10.Training and validation R2 curves for model 3, 7, and 11.

Figure S11.Training and validation Top-3 Accuracy for model 3, 7, and 11.

Figure S12.Training and validation Top-5 Accuracy for model 3, 7, and 11.

Figure S13.Training and validation Top-10 Accuracy for model 3, 7, and 11.

Figure S14.Algorithm 1 flowchart.

Table S4: Model performance based on test data.

Data description:

Table S1: Description of the whole OSA cohort.

Attitude	Value Range (mean)
Female #	8091
Male #	16285
Age	0 – 101 (58.52)
Diff_dgn	0 – 6192 (1121.10)
Visit cost	40 – 1100 (66.27)
Patients’ record length	1 – 515 (8.67)

Table S2: Description of the OSA cohort for cost prediction.

Attitude	Value Range (mean)
Female #	1618
Male #	3337
Age	15 – 94 (59.67)
Diff_dgn	0 – 6095 (1427.47)
Visit cost	40 – 1100 (74.17)
Patients’ record length	4 – 222 (20.67)

Explorative data analysis:

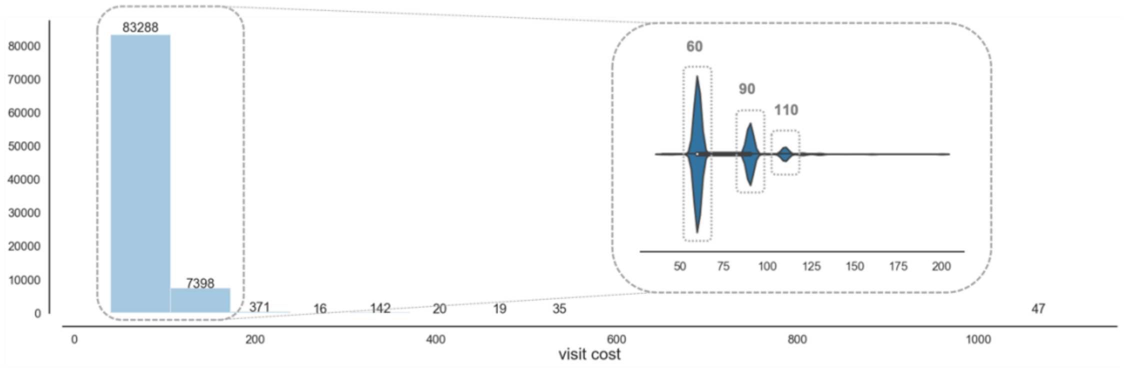

Figure S1. Visit cost distribution.

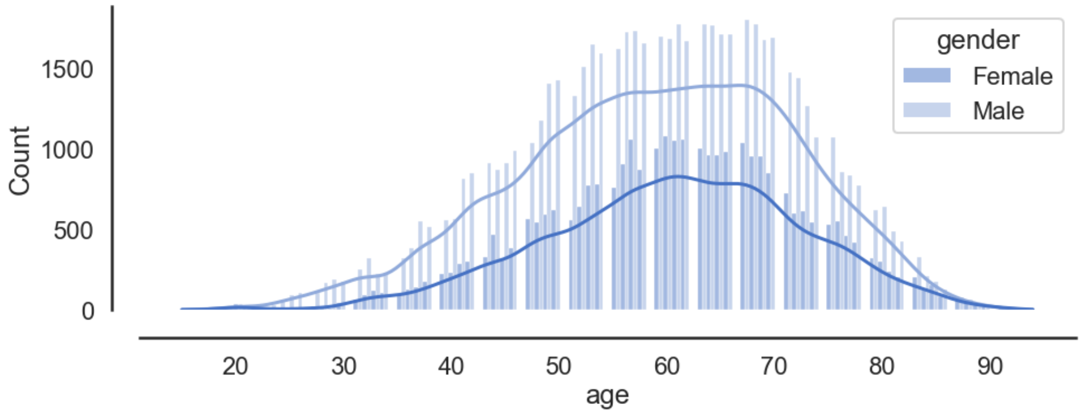

Figure S2. Age distribution for both genders.

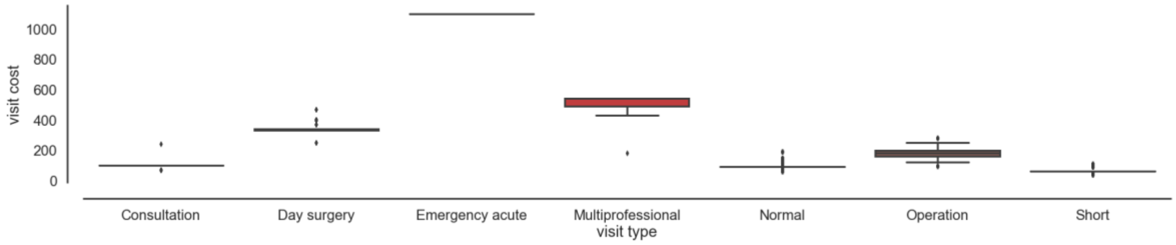

Figure S3. Visit cost distribution for each visit type.

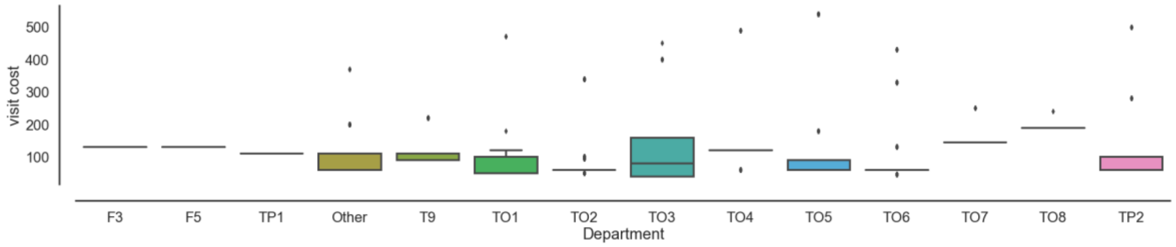

Figure S4. Visit cost distribution for each department.

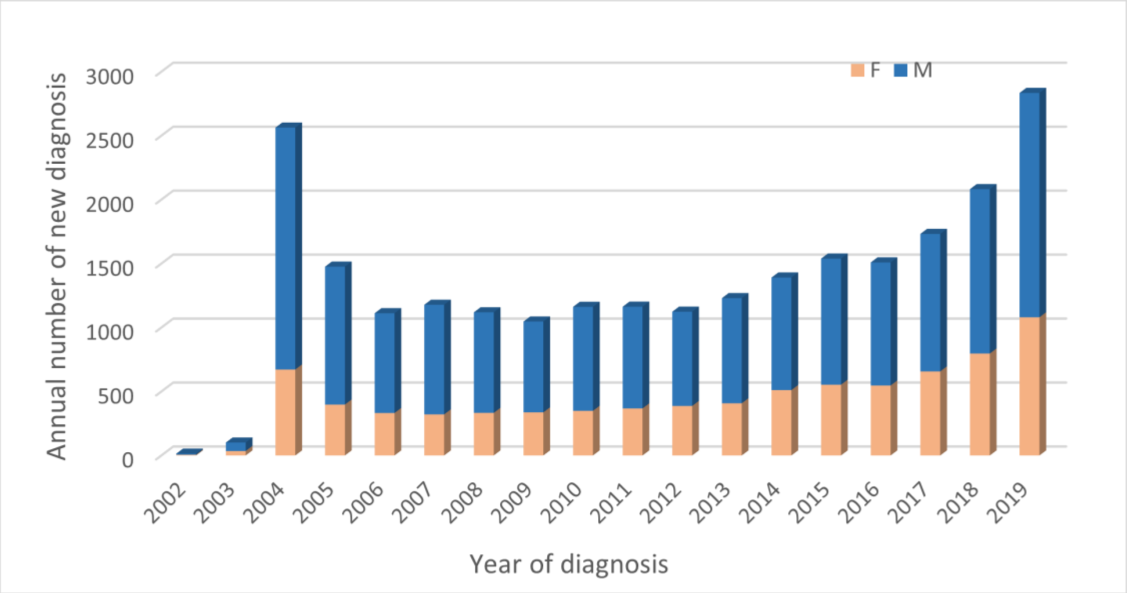

Figure S5. Annual number of new diagonsis for both genders (The legislation in Finland changed in 2004 and long waiting lists were not any more allowed. Therefore the number of new diagnoses peaked in year 2004).

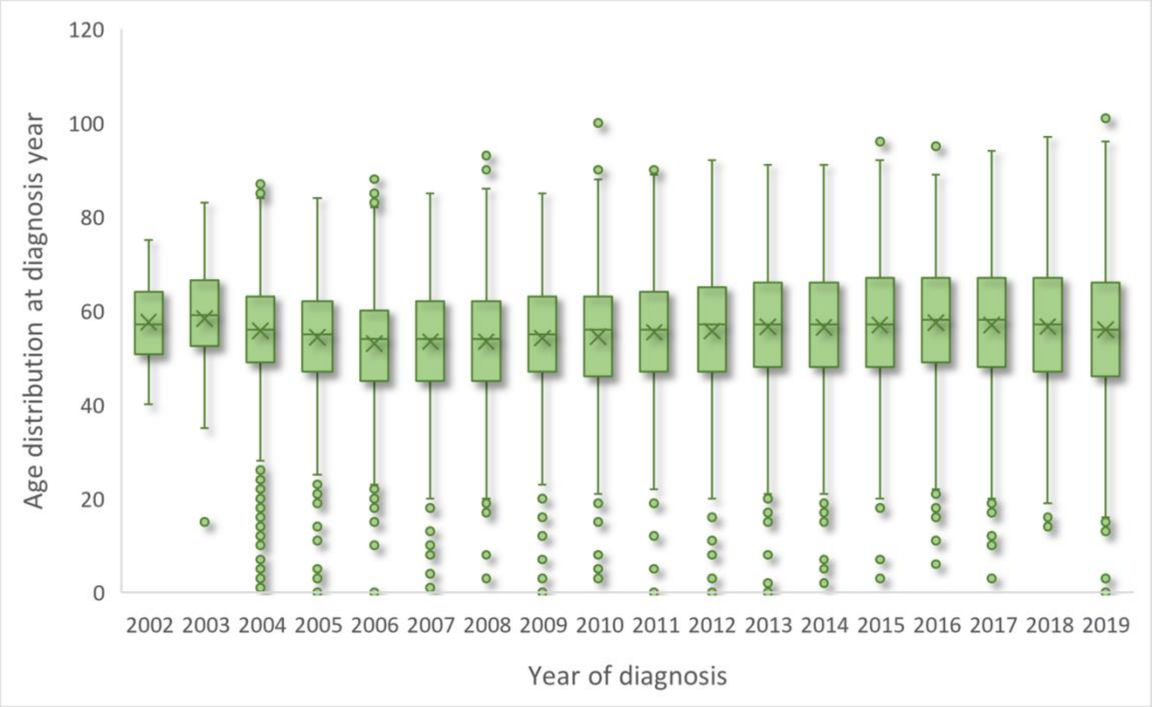

Figure S6. Age distriubution at each year of diagnosis.

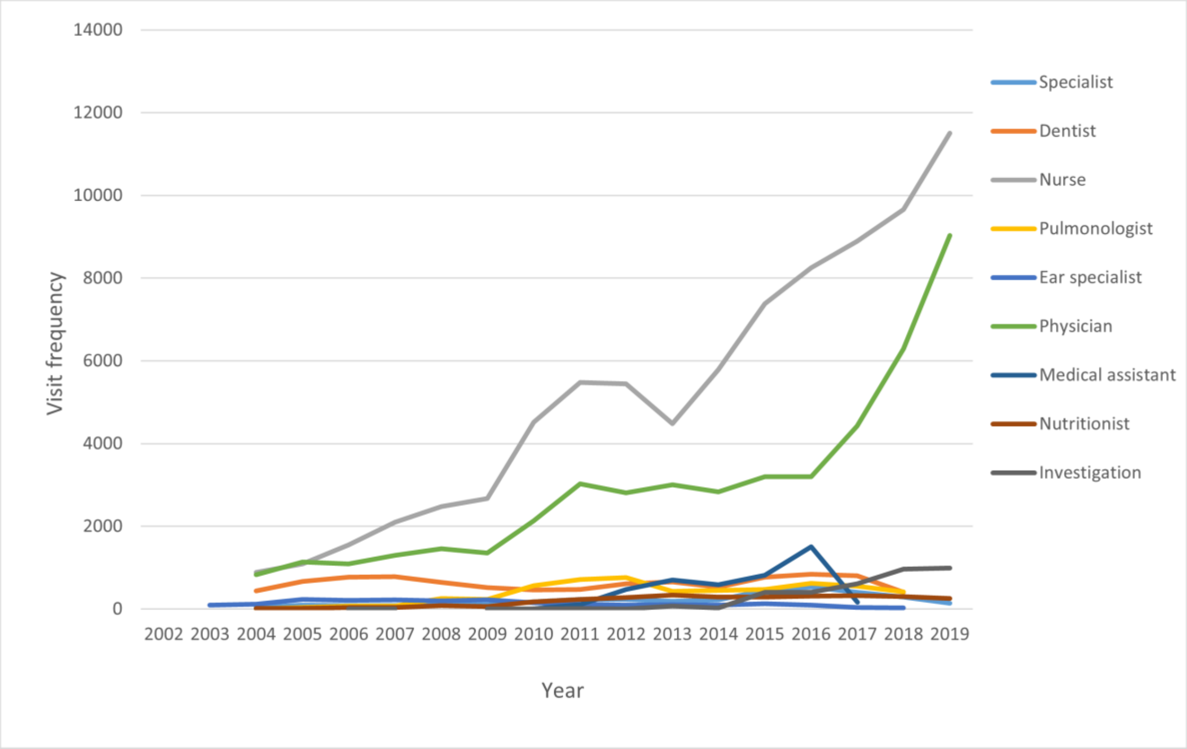

Figure S7. Annual visit frequency for each specialist.

For data augmentation, we filtered patients with more than 4 visits in EHRs. We assume that more than two visits are needed to predict the coming one or two visits. For the cost prediction, we collected the patients with more than 2 visits since at least one visit is needed to predict the next visit.

Model performance with different loss functions and hyperparameters:

The data is first split into three sets: training, validation, and testing. Training, validation, and testing are split into equal parts (80%, 10%, and 10%, respectively). Deep learning algorithms have a lot of parameters, and they need a lot of training data to figure out what these parameters should be set to (Yu et al., 2015). We choose to use 80% of total data for training due to the small size of our cohort, 4887.

Second, we decide to use the Adam optimizer for our research. The word "adaptive moment estimation" appears in its name. A method or procedure for fine-tuning model parameters during training is called an optimizer. As a result, the overall loss can be decreased while improving precision. Adam is our choice since it is suggested as the default optimizer, it is simple to set up, it runs faster and uses less memory than other optimizers, and it needs less adjusting overall. (Gupta, 2022)

Third, we choose to test various combinations of batch size and learning rate to determine which combination best suits the performance of our models. The experiment's learning rates are 0.0001 and 0.0005. The experiment's batch sizes are 64 and 32. There are so a total of 4 combinations.

Fourthly, because there is a significant magnitude difference between L_1 and L_2, we employ three different approaches to merge the two sub loss functions 1) scaling L_1 using common logarithm (log10) and natural logarithm (ln), and 2) calculation of the harmonic mean of L_1 and L_2.

Initial experiments show that L_1 reduces from about 20000 to about 2000, whereas L_2 decreases from about 4 to 0.02. Therefore, we must devise a method for bringing these two losses to a comparable scale of magnitude. We create two scaling plans. Applying logarithm functions to L_1 is the first. Three typical logarithm functions with a range of 2000 to 20000 are depicted in Figure S8 as curves. log2 is the least similar to 4 of the three functions, with a range from 10 to 14. We choose to scale L_1 using the other two. We compare three logarithm functions for the scaling functions and settle on the two previously mentioned ones since log10 and ln can scale the L_1 more effectively.

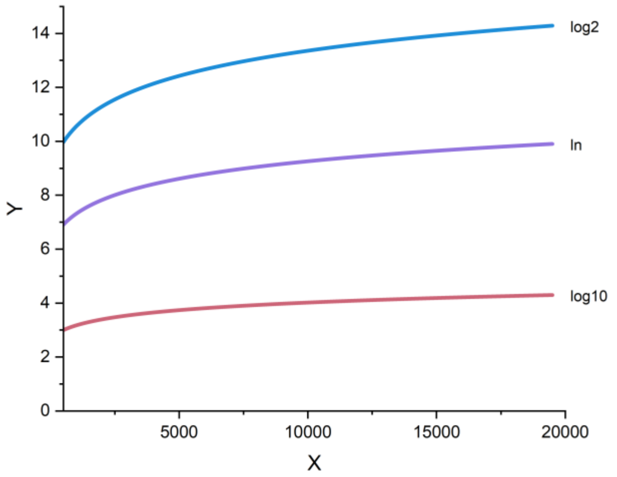

Figure S8. Three common logarithm functions.

Finally, we start training and evaluating Transformer models with 2 encoder layers and 2 decoder layers under different strategies.

Table S3: Model performance based on test data.

Loss Function (L =)	Learning Rate	Batch Size	No.	Indicators for Single Visit Prediction Performance			Indicators for Total Cost Prediction Performance		
				Top-3	Top-5	Top-10	MAE	RMSE	R^2
$\ln(L_1) + L_2$	0.0005	64	1	60.95%	84.26%	93.73%	11.19	134.15	0.797
		32	2	64.28%	83.08%	95.50%	12.94	141.85	0.766
	0.0001	64	**3**	**77.36%**	**87.60%**	**93.77%**	**6.64**	**84.43**	**0.920**
		32	4	77.69%	85.57%	93.64%	9.61	132.67	0.795
$\log10(L_1) + L_2$	0.0005	64	5	78.57%	88.50%	95.46%	10.54	132.60	0.802
		32	6	68.35%	89.06%	96.33%	12.89	141.09	0.768
	0.0001	64	**7**	**85.19%**	**89.11%**	**94.50%**	**7.02**	**92.84**	**0.903**
		32	8	82.73%	84.54%	84.43%	10.80	135.00	0.788
$\frac{2}{\frac{1}{L_1} + \frac{1}{L_2}}$	0.0005	64	9	81.89%	88.65%	95.07%	119.86	238.61	0.358
		32	10	84.05%	91.06%	95.52%	121.48	236.86	0.347
	0.0001	64	**11**	**89.65%**	**95.79%**	**98.13%**	**109.89**	**238.67**	**0.358**
		32	12	82.21%	89.25%	94.34%	125.55	250.88	0.267

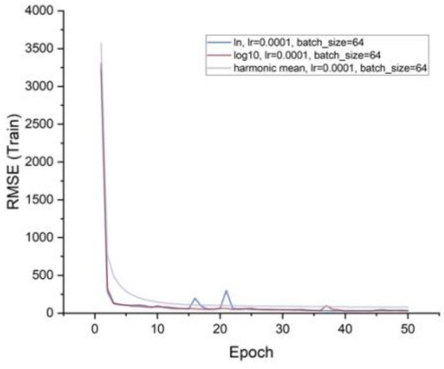

a) Training RMSE

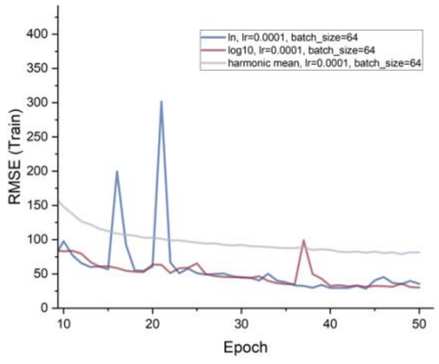

b) Training RMSE from 10th epoch

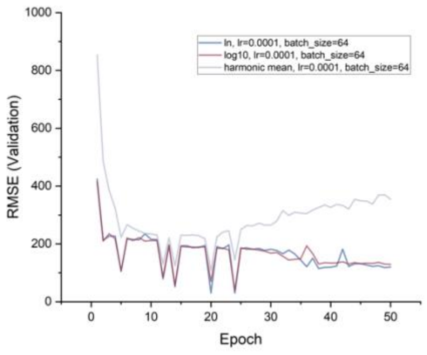

c) Validation RMSE

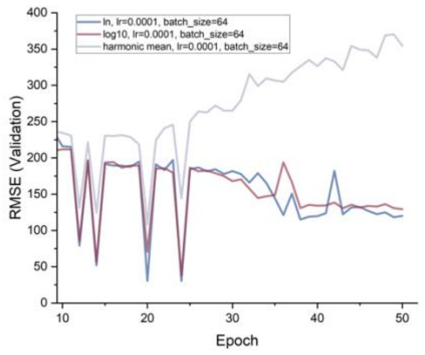

d) Validation RMSE from 10th epoch

Figure S9.Training and validation RMSE curves for model 3, 7, and 11.

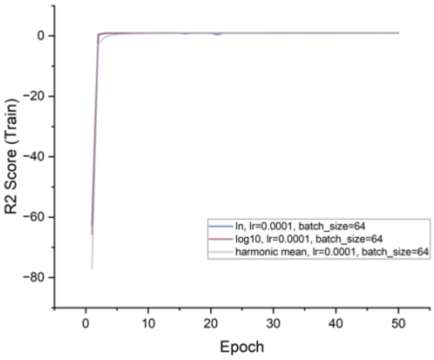

a) Training R^2

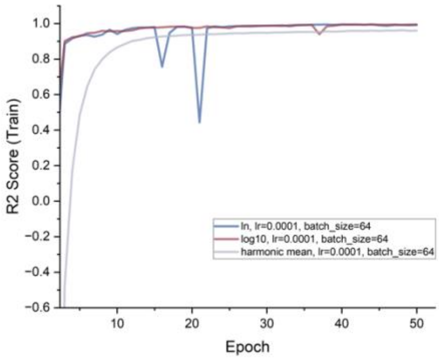

b) Training R^2 from 3rd epoch

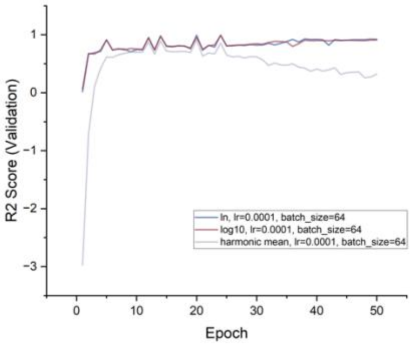

c) Validation R^2

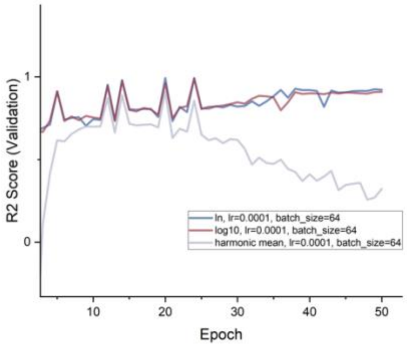

d) Validation R^2 from 3rd epoch

Figure S10.Training and validation R^2 curves for model 3, 7, and 11.

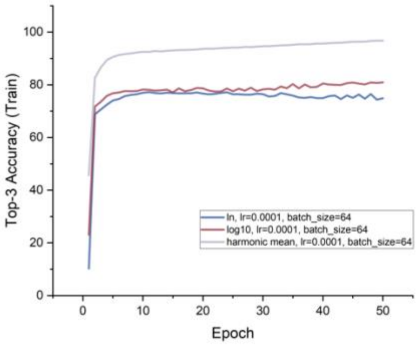

a) Training Top-3 Accuracy

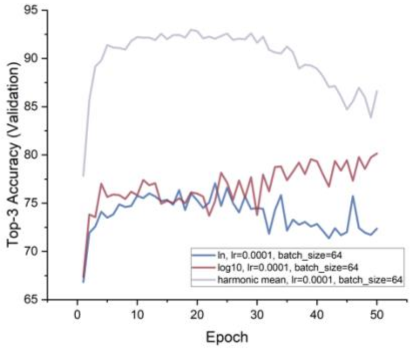

b) Validation Top-3 Accuracy

Figure S11.Training and validation Top-3 Accuracy for model 3, 7, and 11.

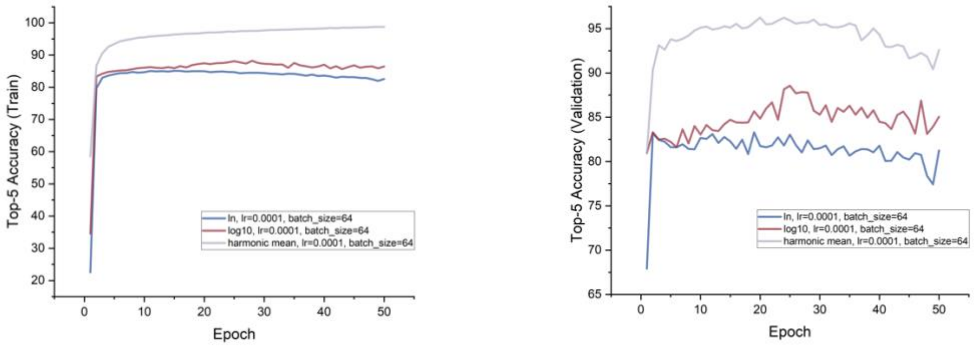

a) Training Top-5 Accuracy b) Validation Top-5 Accuracy

Figure S12.Training and validation Top-5 Accuracy for model 3, 7, and 11.

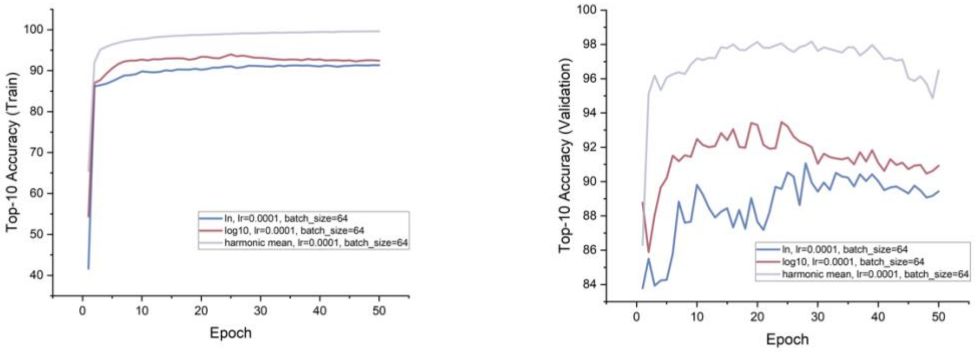

a) Training Top-10 Accuracy b) Validation Top-10 Accuracy

Figure S13.Training and validation Top-10 Accuracy for model 3, 7, and 11.

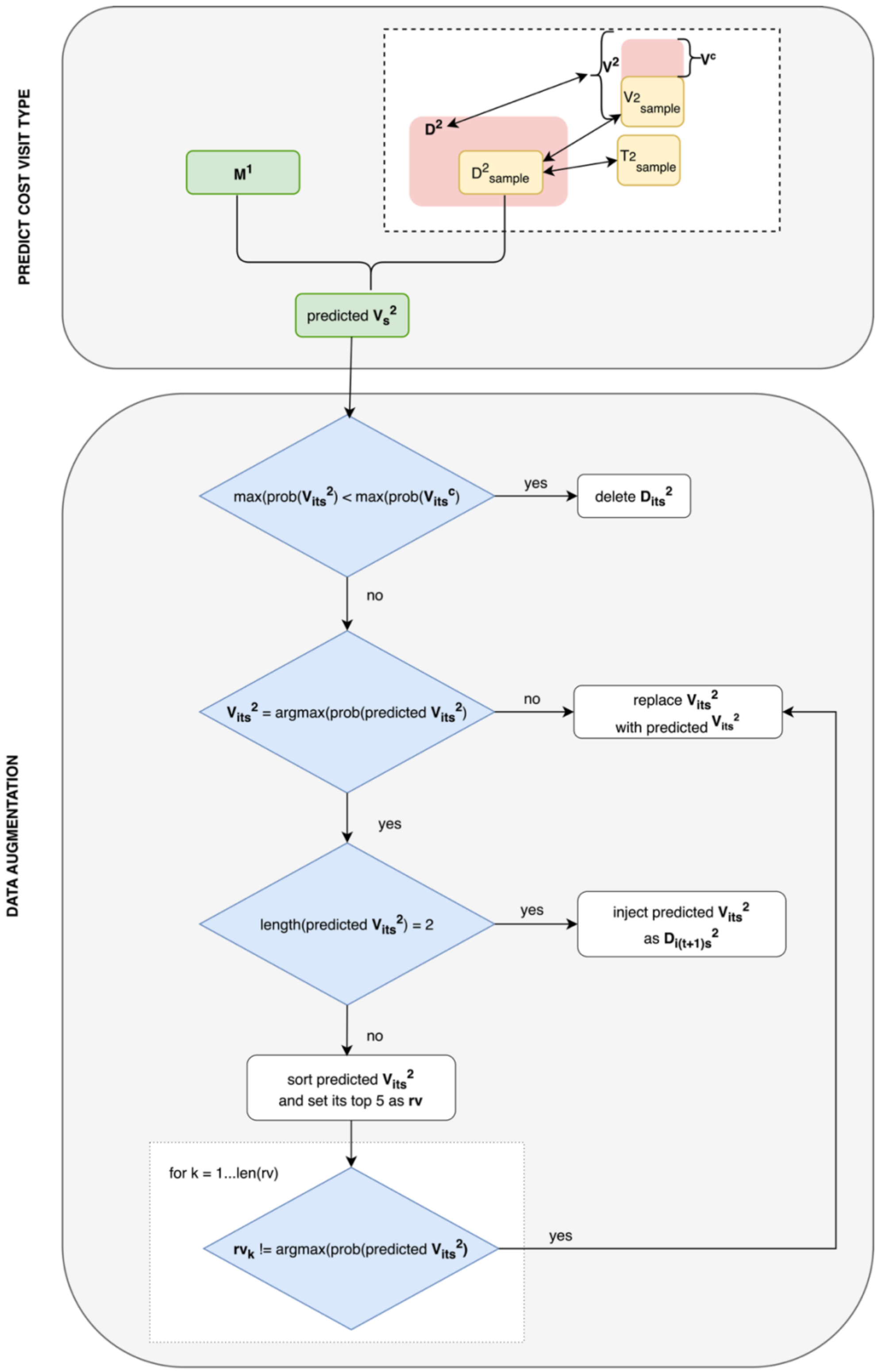

Figure S14. Algorithm 1 flowchart.

For the sake of simplicity, let us assume that D^2 has a visiting vector V = {A, B, C, D, E, F}. When we randomly sample the data, we obtain a visiting vector $V^2{}_s$ = {A, C, D, F}, with its complement being V^c = {B, E}. Now, consider a hypothetical patient who has visited the facility 20 times. We randomly select their first five visits and predict the subsequent visit. By calculating the predicted probability of V, we can compare it to the values of $V^2{}_s$ and V^c. If the maximum value of the probability of $V^2{}_s$ is lower than the maximum value of the probability of V^c, it indicates that the predicted visit is more likely to be outside the set of $V^2{}_s$.

In such a scenario, we would remove the sixth visit from the patient's record. This approach allows us to refine the dataset based on the predictions made by our model.

Computational resource and time:

To achieve better efficiency in running deep learning models, we use a MacBook Pro (version: MacOS Monterey 12.6, memory: 32 GB, chip: Apple M1 Max) as our device and use Anaconda in the version developed for M1. From Anaconda, we then launch the Jupyter Notebook, in which we deploy and run models developed by PyTorch. The computational time for M^1 was two hours and three mins. The computation time for baseline and Transformer M^2 are listed in the following table.

Table S4: Model performance based on test data.

	Original data	With augmented data
LSTM without attention	01h:14m:45s	01h:31m:06s
LSTM with attention	00h:57m:40s	01h:14m:38s
BiLSTM without attention	00h:26m:33s	01h08m:48s
BiLSTM with attention	01h:01m:46s	02h:41m:25s
Transformer	02h:03m:25s	06h:47m:58s

Reference:

Gupta, A. (2022). A Comprehensive Guide on Deep Learning Optimizers. Retrieved November 7, 2022, from https://www.analyticsvidhya.com/blog/2021/10/a-comprehensive-guide-on-deep-learning-optimizers/

Yu, F., Seff, A., Zhang, Y., Song, S., Funkhouser, T., & Xiao, J. (2015). LSUN: Construction of a Large-scale Image Dataset using Deep Learning with Humans in the Loop. https://doi.org/10.48550/arxiv.1506.03365
